# Communicative impairment and its neural correlates in Alzheimer's disease and frontotemporal dementia

**DOI:** 10.1002/brb3.3420

**Published:** 2024-03-17

**Authors:** Alexa Haeger, Janka Muising, Sandro Romanzetti, Bruno Fimm, Oliver Matz, Jörg B. Schulz, Stefan Heim, Kathrin Reetz

**Affiliations:** ^1^ Department of Neurology RWTH Aachen University Aachen Germany; ^2^ JARA‐BRAIN Institute Molecular Neuroscience and Neuroimaging Forschungszentrum Jülich GmbH and RWTH Aachen University Aachen Germany; ^3^ Institute of Neuroscience and Medicine (INM‐1) Research Centre Jülich Jülich Germany; ^4^ Department of Psychiatry, Psychotherapy and Psychosomatics, Medical Faculty RWTH Aachen University Aachen Germany

**Keywords:** *Aachener KOMPASS*, Alzheimer's disease, communication, communication barriers, frontotemporal dementia, primary progressive aphasia, aphasia, MRI, speech and language therapy

## Abstract

**Objective:**

Communication skills can deteriorate in neurodegenerative diseases such as Alzheimer's disease (AD) and frontotemporal dementia (FTD); however, their clinical assessment and treatment in patient care can be challenging. In the present study, we aimed to quantify the distinctive communication resources and barriers reported by patients and their relatives in AD and FTD and associated these communicative characteristics with clinical parameters, such as the degree of cognitive impairment and atrophy in language‐associated brain areas.

**Methods:**

We assessed self‐reported communication barriers and resources in 33 individuals with AD and FTD through an interview on daily‐life communication, using the *Aachener KOMPASS* questionnaire. We correlated reported communication barriers and resources with atrophy from high‐resolution 3T brain magnetic resonance imaging, neuropsychological assessment, and neurodegenerative markers from cerebrospinal fluid.

**Results:**

Communicative impairment was higher in FTD compared to AD. Increased reported communication barriers in our whole sample were associated with the atrophy rate in the left middle temporal lobe, a critical site within the neuronal language network, and with depressive symptoms as well as the semantic word fluency from neuropsychological assessment. The best model for prediction of communicative impairment included the diagnosis (AD or FTD), semantic word fluency, and depressive symptoms.

**Conclusions:**

Our study demonstrates that communication barriers and resources can be successfully assessed via a structured interview based on self‐report and report of patients’ relatives in practice and are reflected in neuroimaging specific for AD and FTD as well as in further clinical parameters specific for these neurodegenerative diseases. This can potentially open new treatment options for clinical practice and patient care.

## Graphical abstract

Communication can be impaired in dementia, leading to a reduced quality of life of both affected patients and their relatives. We show that communicative barriers and resources can be successfully assessed via a structured interview in dementia during clinical routine and that communicative barriers are associated with temporal brain atrophy, depressive symptoms, and semantic word fluency from neuropsychological assessment. Perspectively, evaluation of individual communication barriers and resources can strengthen therapeutic interventions.

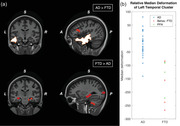

## 1 INTRODUCTION

Verbal communication is essential for sharing human needs and emotions and for succeeding in social interaction. It is also an important preventive factor for cognitive decline (Clair, 2016; Hari & Kujala, 2009; Livingston et al., 2020). Individuals with cognitive decline often face speech and language impairments due to an affected neuronal language network, leading to difficulties in word finding, word retrieval, word anomia, and the articulation of words (Klimova & Kuca, 2016). This can result in restricted communication skills with a negative impact on the quality of life, possibly leading to social isolation and further clinical deterioration (Klimova & Kuca, 2016; Potkins et al., 2003).

Depending on the etiology of the cognitive impairment, for example, in Alzheimer's disease (AD) and frontotemporal dementia (FTD), communication can be impaired in different cognitive–linguistic domains, including word finding, comprehension, storytelling, semantics, and pragmatics (Ash et al., 2006; Heim et al., 2020; Jootun & McGhee, 2011; Mathuranath et al., 2011; Rousseaux et al., 2010). Already in the early stages of AD, difficulties in word finding and storytelling can occur and later progress to the incapability to participate in daily conversations due to increasing cognitive decline, reflected by structural cerebral changes such as temporal and hippocampal atrophy (Klimova et al., 2015). The behavioral variant of FTD (bvFTD) is often accompanied by personality and behavioral changes, leading to reduced speech output, reduced initiation in conversations and echolalia (Blair et al., 2007; Frank, 1994). Primary progressive aphasia (PPA) variants most frequently occur in FTD, comprising a semantic variant (svPPA), an agrammatic variant (nf‐avPPA), and a logopenic variant (lvPPA), with the last also often occurring in AD (Bang et al., 2015; Grossman, 2010; Mesulam et al., 2014).

Despite its clinical importance, there is still a lack of eligible assessment tools for functional communication in individuals with dementia, reflecting their self‐reflected needs, feelings, preferences, and communicative barriers in daily life (ASHA, 2019). Maintaining communication skills and compensating for limitations in communication are important aspects of the patients’ quality of life. Since speech therapy needs to be individualized and person‐centered, clinical assessment of the degree of impairment is essential (e.g., Bur et al., 2019). Existing tests such as the *Aachen Aphasia Test* (AAT) (Huber et al., 1983; Willmes et al., 1983), the *Functional Communication Therapy Planner* (FCTP) (Worrall, 1999), the *Bayer Activities of Daily Living Scale* (B‐ADL) (Hindmarch et al., 1998), the *Assessment for Living with Aphasia* (ALA) (Kagan, 2011; Simmons‐Mackie et al., 2014) as well as the *Progressive Aphasia Severity Scale* (PASS) (Henry et al., 2018) were designed to assess and quantify acquired aphasic disorders mainly after a stroke; therefore, they show limitations in capturing the language‐related symptoms specific to dementia. Furthermore, the association of such assessment tools with disease‐specific parameters is also lacking. Studies indicate that social networks shrink with age, limiting interactions with relatives, friends, and acquaintances (Prahl & Schroeter, 1996; Wagner et al., 1996). Mobility restrictions reduce peer interactions, and neighborhood relationships often become key communication channels. Leisure activities, like handiwork, housework, writing, reading, and solving puzzles, are prevalent among older adults. Media consumption, including reading newspapers, listening to the radio, and watching TV, structures daily life and provides information and entertainment. The telephone is a vital tool for maintaining contact with others due to its ease and convenience, and the use of computers is increasing for similar reasons (Schulze, 1998). When regarding communication in dementia, not only do speech and language impairments occur due to the neurodegenerative process, but also changes in social interaction, daily activities, and usage of media. Their overall assessment can help in identifying resources and barriers which can then be considered in therapeutic approaches. For example, some people with cognitive impairment might prefer the usage of a phone or a computer for communication whilst others might set a focus on personal interaction. Therapists could therefore further strengthen and encourage communicative competences as resources, while also helping to overcome barriers when assessing those by an appropriate tool. In this context, the *Aachener KOMPASS* (Dretzko & Lehmann, 2012; Dretzko et al., 2013) is a German questionnaire aiming to assess the functional communication skills and the communicative behavior of elder persons (over 60 years), revised in 2017 and adapted for application in dementia (Heim, 2020; Rembeck, 2017). It takes into account the reduced resilience and attention span of individuals with dementia. This tool can be used to assess self‐reported functional communication, as well as to identify individual communication barriers and resources.

In the present study, patients with AD and FTD were examined via the revised version of the *Aachener KOMPASS* to assess self‐reported communicative impairment, reflected in both communication barriers and communication resources related to each of these neurodegenerative diseases.

As an underlying goal of this study, we aim to examine whether communication barriers and resources can be successfully assessed via self‐report of patients and relatives in the form of a structured interview and to further characterize the features of communicative impairment in AD and FTD. Additionally, we examine to which extent these features are reflected in disease stage, that is, atrophy levels detected by magnetic resonance imaging (MRI) and cognitive and neuropsychiatric impairment. We expected that communicative impairment would be associated with the degree of disease‐specific atrophy patterns and cognitive impairments reflecting disease progression. We also anticipated a correlation between neuropsychiatric symptoms, such as depressive symptoms, and the level of communicative impairment. By assessing communicative impairment, we could address identified barriers and bolster resources, as indicated by patients and their relatives, in individualized therapeutic interventions, alleviating the disease burden in daily interactive life in dementia.

## 2 MATERIAL AND METHODS

### 2.1 Study design and setting

We performed a prospective study in patients with AD and FTD who were recruited from the Memory Clinic of the Department of Neurology, RWTH Aachen University. The study project was approved by the local ethics committee of the RWTH Aachen University (EK 083/15) and was carried out in accordance with the latest version of the Declaration of Helsinki. All participants gave written informed consent prior to participating.

### 2.2 Participants

**FIGURE 1:**
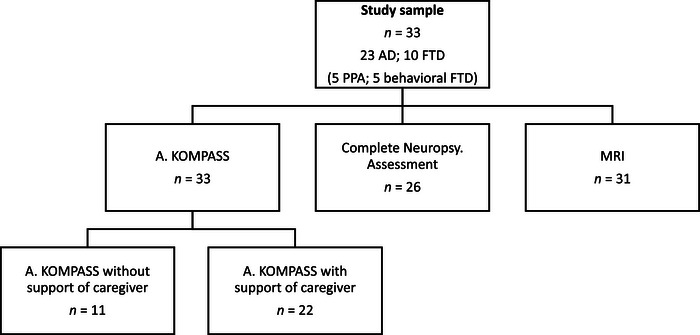
Overview of the study sample, including participants with Alzheimer's disease (AD) and frontotemporal dementia (FTD). This figure illustrates their completion of *Aachener KOMPASS* (A. KOMPASS), magnetic resonance imaging (MRI), and neuropsychological assessment (Neuropsy. Assessment). PPA, primary progressive aphasia. Of the subjects, 26 completed the entire neuropsychological assessment.

A total of 33 participants (23 AD, 10 FTD, range 57−78 years; mean age in the AD group: 70.4 years; including 7 females; mean age in the FTD group: 66.3 years; including 5 females; mean age of the entire sample: 69.2 years) were included in the study. The AD diagnosis followed international research criteria for dementia, according to the National Institute on Aging and Alzheimer's Association (NIA‐AA) criteria (Jack et al., 2018; McKhann et al., 2011). The PPA variant of FTD was diagnosed based on clinical and imaging parameters as detailed in Gorno‐Tempini et al. (2011), and the behavioral variant FTD was evaluated through behavioral parameters and neuroimaging (MRI and FDG‐PET) as described in Rascovsky et al. (2011). The FTD sample was composed of two subgroups: five participants with bvFTD and five with diagnosed PPA. Within the PPA‐FTD subgroup, three had svPPA and two had nf‐avPPA. For a more detailed clinical description of the FTD sample, see Table S1. In the AD group, seven participants were in the prodromal stage of the disease, and 16 were in the mild to moderate stages, as determined by the Mini‐Mental State Examination (MMSE) scores according to the guidelines of the National Institute for Health and Care Excellence (NICE) (prodromal stage: 30−27 out of 30 points; mild stage: 26−21 out of 30 points; moderate stage: 20−10 out of 30 points) (Folstein et al., 1975). One participant with AD was diagnosed with lvPPA. Figure 1 provides an overview of the entire study sample. Given our focus on specific group differences between AD and FTD, we did not include a cognitively healthy control group. To ensure that results were not influenced by our prodromal AD patients, control analyses were additionally performed with exclusion of the six AD patients with an MMSE ≥28 points.

### 2.3 Procedures

The study participants underwent cognitive, neuropsychiatric, and communication assessments as well as MRI measurements at 3T magnetic field as detailed below. For diagnosis at our memory clinic, participants had previously undergone a lumbar puncture for the evaluation of cerebrospinal fluid (CSF) markers. The analysis was performed at the Neurochemical Laboratory at the University of Göttingen, with normal ranges specified as follows: amyloid ß1–40, amyloid ß1–42 > 450 pg/mL, amyloid ß1–42/40 ratio > 0.5, total tau < 450 pg/mL, and phosphorylated tau < 61 pg/mL) (Reiber, 2005; Zettl et al., 2005).

### 2.4 Communication assessment

To identify daily‐life communication resources and barriers, we applied a shortened version of the *Aachener KOMPASS*, adapted for individuals with dementia. The original *Aachener KOMPASS* (Dretzko & Lehmann, 2012, 2013) is a comprehensive questionnaire comprising 28 closed‐ended and 6 open‐ended questions. These questions address communicative behavior, media use, writing and reading habits, and include biographical information for individuals over 60. The questionnaire encompasses various aspects of communication, such as different communication media (e.g., written, spoken, and electronic), types of communication partners (e.g., relatives, medical staff, and neighbors), conversation topics (e.g., hobbies, weather, and health issues), and difficulties encountered in communicative situations (e.g., environmental noise, following complex arguments, word retrieval, and so on). Participants also have the opportunity to express their preferences for changes in communicative behavior through the open‐ended questions. Aligned with the *International Classification of Functioning, Disability and Health* (ICF) (Üstün et al., 2003), the questionnaire is therefore divided into three main parts: (I) Communicative activities, (II) resources, and (III) communication barriers, covering the mentioned four areas of conversation management, reading, writing, and media use. In the closed‐ended questions (1–28), there are 3 to 17 answer options (items) available, with participants able to select multiple answers. Questions 1–22 also allow respondents to rate selected answers for importance, frequency, or difficulty on a four‐level scale. This approach helps differentiate between relevant and less relevant responses, emphasizing significant areas of the patient's life. Difficulty level assessment inquires about an individual's resources and barriers; a reported difficulty indicates a barrier, while ease suggests a resource. Biography questions (23–28), which include inquiries about personality traits, are positioned at the end to gather biographical information relevant for therapy.

The original *Aachener KOMPASS* was adapted in 2017 for use with people with cognitive impairment, resulting in a shorter version with 17 closed‐ended and 3 open‐ended questions (Heim, 2020; Rembeck, 2017). During the assessment, the questionnaire is provided in both written and spoken formats, and supporting sketch images may be shown for each question. This allows the *Aachener KOMPASS* to be used both as a questionnaire and as a structured interview guideline by speech–language pathologists, who can read out the questions to the patient, potentially in the presence of caregivers. In our study, 22 participants completed the communication assessment with caregiver assistance. When participants were unable to answer a question, the caregivers provided their input. The time taken to complete the entire assessment was recorded.

The *Aachener KOMPASS* was analyzed as follows: The answers were categorized into communication resources (derived from parts I and II) and communication barriers (derived from part III), based on their frequency of occurrence. Activities requiring communication, such as making phone calls, writing letters, meeting with family and friends, or arranging doctor's appointments, were rated as a resource if answered with “yes.” If a question about a specific activity also involved assessing difficulty level, it was counted as a resource if indicated as “rather easy” and as a barrier if rated as “rather difficult” (refer to Table 1). Multiple‐choice questions were categorized into barrier or non‐barrier and resource or non‐resource questions, depending on the participant's response. For example, for the question “Do you struggle during conversations?,” participants could answer “yes” or “no.” A “no” response was considered a communicative resource, while a “yes” response required further clarification through additional statements, such as “I find it difficult to understand what others are saying during conversations” or “I forget what I wanted to say,” pointing to a communication barrier. Given statements could then be used to plan therapeutic goals. Responses to open‐ended questions were also categorized as barriers or resources, depending on the type of response. An example of an open‐ended question from the *Aachener KOMPASS* is “Have there been any changes in your daily communication in the last few years?” A participant's response such as “Yes, I do not talk to a lot of people because I cannot express what I would like to tell them” would be categorized as a “yes” response and a communication barrier, due to the nature of the answer relating to an impairment.

**TABLE 1:** Items from the *Aachener KOMPASS* featuring selected questions and items used for calculating the *resources and barriers scores* with additional explanations on the questions; open‐ended questions are marked with an asterisk (*). Responses not relevant for the evaluation of the *barriers score* for items 1–3 are in gray text color.

Resources score			
Items	Questions and explanations	Possible answers (multiple choice)	Personal evaluation
1	Which of these people do you talk to regularly? ➔ Number of conversation partners as an indicator of communicative skills and resulting social participation.	Acquaintances Doctor Friends Neighbors Children Spouse/partner Other family members Contacts during shopping Grandchildren/great‐grandchildren Home help Caregiver/nurse/therapist	**Importance of these conversations** *Rather important* *Rather unimportant*
2	What do you write in your free time/daily life? ➔ Written language as a communicative resource.	Birthday/Christmas cards Crossword puzzles/sudoku Letters E‐mails Text messages	**Importance of these forms of writing** *Rather important* *Rather unimportant*
3	Where in everyday life do you rely on writing? ➔ Using written language as a resource for future therapy approaches and as an indicator of independence through communication.	Shopping lists Calendar entries Notes Cooking recipes Bank transactions Forms/letters/E‐mails	
4	What do you read in your free time/in your everyday life? ➔ Written language as a communicative resource.	Books Reference books/trade journals Magazines Newspapers Encyclopedias Letters E‐mails Postcards	**Importance of these forms of reading** *Rather important* *Rather unimportant*
5	Where in everyday life do you rely on reading? ➔ Using written language as a resource for future therapy approaches and as an indicator of independence through communication.	Reading aloud for others Shopping Household Cooking recipes Package inserts for medication City maps Timetables Invoices Formalities	
6	What media do you use? ➔ Use of different media as a resource: possible approaches for speech therapy, as media such as cell phone use and Internet use can serve to compensate for barriers.	Telephone Mobile phone Newspaper Radio Television Internet Social media	**Importance of usage of the media** *Rather important* *Rather unimportant*
7	What hobbies do you have? ➔ As an indicator of social participation and as possible topics for speech therapy in order to design patient‐oriented therapy content.	Painting Gardening Workshops Museum/theater/concert visits Cooking Walking Board games Crafts Cars/motorcycles Technology Traveling Dancing Music Sports Clubs	**Importance of conversations in these hobbies** *Rather important* *Rather unimportant*
8	In what situations do you have conversations? ➔ Number of different communicative situations in everyday life as an indicator of communicative resources.	Meetings with the family Meetings with friends When looking after family members Within the church community At the doctor When shopping At the hairdresser/beauty salon In a restaurant While spending time with therapists/caregivers On bus and train journeys	**Importance of conversations in these situations** *Rather important* *Rather unimportant*
9*	Has the way you spend your free time changed in the last 5 years? If yes, how? What would you like to do more often again? ➔ Indicator for social participation and independent social interaction.	*Open answers*	

Barriers score			
Items	Questions and explanations	Possible answers (multiple choice)	Personal evaluation
1	When do you use the telephone? ➔ Communication with strangers often described as a communicative challenge by the participants.	• *Organizational matters with strangers (doctor, health insurance)* • Conversations with the family • Conversations with friends and acquaintances	**Importance of telephone** *Rather important* *Rather unimportant*
2	Which statements apply to you? ➔ Large group size as an indicator of social interaction and participation.	• *I have conversations in groups of more than four people* • I have conversations with two people • I have conversations in groups of two to four people	**Importance of conversations of this kind** *Rather important* *Rather unimportant*
3	How do you feel about conversations with the following people? (acquaintances/ strangers) ➔ Communication outside of family and friends as an indicator of safety in communication.	• *With acquaintances* • *With strangers* • In the immediate family circle • With friends • With caregiver/nurse/therapist	**Level of difficulty** *Rather easy* *Rather difficult*

Barriers score		
Items	Questions and explanations	Personal evaluation
	**How do you feel about the following when speaking?**	**Level of difficulty**
4	Resolving misunderstandings in conversations is…?	*Rather easy* *Rather difficult*
5	Changing the subject in conversation is …?	*Rather easy* *Rather difficult*
6	Engaging in discussions is…?	*Rather easy* *Rather difficult*
	**How do you feel about the following when listening?**	**Level of difficulty**
7	Listening when people are talking fast is…?	*Rather easy* *Rather difficult*
8	Listening when topics are not familiar is…?	*Rather easy* *Rather difficult*
9*	Has anything changed in your communication behavior in the last five years? If yes, what? ➔ Changes in communication behavior as an indication of communicative barriers.	*Open answers*

For the assessment of the communication barriers, nine items demonstrating the largest variability in responses among participants were chosen for scoring. The *barriers score* for each participant was calculated as the sum of these items, ranging from 0 to 9 points. Similarly, for the communication resources, the nine most frequently indicated items were selected to form the *resources score*. High scoring numbers in the *barriers score* indicated more communication barriers, just as high scores in the *resources score* indicated more communication resources (see also Table 1). These scores were used for further data analysis. Five questions (four multiple‐choice questions and one open‐ended) were excluded from the analysis, as they were not answered by all participants and thus not deemed relevant by each individual.

### 2.5 Neuropsychological assessment

All subjects underwent neuropsychological assessments, which included the MMSE, the *Montreal Cognitive Assessment* (MoCA) (Nasreddine et al., 2005), and semantic word fluency from the *Consortium to Establish a Registry for Alzheimer's Disease* (CERAD Plus) (Morris et al., 1989). MMSE scores were utilized as an indicator of the severity of dementia according to the NICE guidelines. Additionally, the Boston Naming Test, and Word List Learning and Recall from the CERAD Plus battery were administered. To assess depressive symptoms, the 21‐item *Beck Depression Inventory* (BDI‐II) (Beck et al., 1996) was used.

### 2.6 3T MRI and analysis

Study participants underwent MRI acquisitions using a 3T Prisma scanner (Siemens) at the RWTH Aachen University Hospital. Brain atrophy was assessed using a high‐resolution T1‐weighted MPRAGE sequence (TR = 2400 ms; TI = 1000 ms; TE = 2.36 ms; Acquisition matrix = 288 × 288; Resolution = 0.8 mm^3^). Twenty‐nine participants had their MR and AK assessments conducted within the same month. However, two participants’ health conditions precluded concurrent MRI scanning; thus, their MRIs from 1 year earlier were used in the analysis (as shown in Figure 1). Two individuals, one with bvFTD and one with svPPA, were unable to undergo MRI due to contraindications. In total, 31 MRIs were analyzed using MATLAB and Advanced Normalization Tools (ANTs) (Avants et al., 2009). T1 images were segmented into gray matter, white matter, CSF, and brain lobes such as frontal, temporal, limbic, parietal, and occipital lobes for left and right hemisphere in ANTs. For extraction of hippocampal volume, VolBrain segmentation was applied, and volumes for both the left and right hippocampus were reported (Manjón & Coupé, 2016). A voxel‐based‐morphometry (VBM) analysis was conducted to identify morphometric differences between AD and FTD groups and to associate these with clinical parameters. This was done by creating deformation maps from the individual log‐Jacobian images. The template for the VBM was taken from *miccai2012‐multi‐atlas‐challenge‐data*, its original MRI scans coming from *OASIS* (https://www.oasis‐brains.org/). Permutation analysis on the deformation maps (*n* = 2000 permutations; *p* < .05) including age and sex as covariates was then performed with BROCCOLI (Eklund et al., 2014) and cluster results reported at an uncorrected and at cluster corrected *p* < .05 level. Clusters identified as statistically significant were used to correlate the degree of volumetric reduction with speech impairment. This was performed by extracting voxel‐wise parametric values inside the mask from the deformation maps and performing correlation on the median of the extracted deformation data. The median was used for consideration of the non‐Gaussian distribution of the deformation values. Additional volumetric comparisons were performed on temporal, parietal, limbic, and frontal lobes as well as the hippocampi, as regions of interest.

### 2.7 Definition of objectives

The primary endpoint of the study was to evaluate differences in communicative impairment, specifically the communication *barriers and resources score*, between the AD and the FTD groups.

The secondary endpoints included an analysis of the association between communicative impairments and cognitive decline, as well as neuropsychiatric symptoms, particularly symptoms of depression. Additionally, we compared the communication scores of the two groups in relation to atrophy patterns identified in the MRI group analysis and the neurodegeneration markers obtained from CSF.

### 2.8 Statistical analysis

Statistical analysis of the data was conducted using the Statistics Package for the Social Sciences (SPSS) Version 25 (IBM Corp., 2017), MATLAB (Version R2019b), and R (Version 1.1.463). The study groups were subjected to exact Fisher's tests and Mann–Whitney U tests with reporting *p*‐values and *z*‐values for comparative analysis. Cohen's *d* was calculated to determine effect sizes, categorizing *d* = 0.2–0.5 as low, 0.5–0.8 as moderate, and >0.8 as large effect. Spearman's correlation coefficients (*r*) were used to report correlation analyses. Multiple regression analysis was performed using leave‐one‐out cross‐validation (LOOCV), with bias‐corrected root mean square errors (RMSE) and ANOVA results from the multiple regression analysis used for model evaluation. A total of 29 subjects were included in the multiple regression analysis; those with missing values were excluded. This included one subject without MRI, one without MoCA, one without BDI‐II and one without MoCA, MRI, and BDI‐II. All statistical analyses considered a significance level of *p* = .05. Correction for multiple comparison was performed via Bonferroni–Holm test for group comparisons of MRI parameters. Given the exploratory nature of this study, results derived from the *Aachener KOMPASS* and its association with neuronal parameters were not corrected for multiple comparisons.

## 3 RESULTS

### 3.1 Clinical characteristics of the AD and FTD groups

In the cognitive short screening tests, the MoCA and MMSE, no significant differences were observed between the AD and the FTD group. Similarly, the BDI‐II results showed no significant differences in depressive symptoms between both groups. However, the FTD group exhibited lower semantic word fluency compared to the AD group (*p* = .0041; *zval* = 2.87; *d* = 1.23).

Neurodegenerative markers in CSF showed typical amyloid and tau pathology in the AD group. Concerning segmentation, that is, atlas‐based region of interest analyses on MRI results, the FTD group revealed lower left temporal brain volumes and higher right parietal volumes compared to the AD group. No significant difference was found in total hippocampal volume between the two groups.

An overview of the detailed results and the corresponding *p*‐values is given in Table 2.

**TABLE 2:** Demographic and clinical data of participants with Alzheimer's disease (AD) and frontotemporal dementia (FTD). *p*‐values and *z*‐values from the Mann–Whitney U‐test are shown in bold when *p* < .05. The table presents the median and range of results (in brackets); It also includes an illustration of volumetric region‐of‐interest differences, visual MR scoring, and cerebrospinal fluid results. Regional volumetric results are adjusted for total intracranial volume. Gray matter encompasses both cortex and deep gray matter. Significant *p*‐values of volumetric magnetic resonance imaging results and MR scorings as well as CSF results surviving Bonferroni–Holm correction are marked in bold as ** when significant at *p* < .001. Reference CSF values, obtained from the Neurochemical Laboratory at the University of Göttingen, include: tau (<450 pg/mL), phospho‐tau (<61 pg/mL), amyloid 1–42 (>450 pg/mL), amyloid ß1–42/40 ratio (>0.5); For Boston Naming test: *n* = 28/33 subjects—Data missing for 3 AD patients due to organizational reasons, and exclusion of 2 FTD patients due to difficulty understanding test instructions; For the Word List Learning Task: *n* = 27/33 subjects—Data missing for 3 AD patients due to organizational reasons and exclusion of 3 FTD patients due to difficulty understanding test instructions.

	AD	FTD	*z*‐value	*p*‐value
**Demographics**				
**Study participants *n***	23	10		
**Age [years]**	70 (61–78)	66 (57–78)	1.22	.22
**Education [years]**	11 (8–21)	10.5 (8–20)	1.52	.13
**Sex [m/f]**	16/7	5/5	1.03	.30
**NPSYCH and A. KOMPASS**				
**Duration AK [min]**	25 (15–37)	31.5 (20–52)	**2.06**	**.039**
**Barriers score**	3 (0–7)	7 (1–9)	**3.17**	**.0015**
**Resources score**	7 (5–9)	6.5 (0–9)	1.49	.14
**MMSE [/30]**	25 (18–30)	23 (0–30)	0.90	.37
**MOCA [/30] (*n* = 32)**	21 (11–27)	17.5 (0–28)	1.87	.061
**BDI‐II [/63] (*n* = 31)**	6 (0–23)	12 (1–29)	1.40	.16
**Semantic fluency (*n* = 33)**	14 (6–24)	7 (0–23)	**2.87**	**.0041**
**Boston naming (*n* = 28)**	15 (12–15)	13 (4–15)	**2.81**	**.0049**
**Word list learning (*n* = 27)**	12.5 (2–26)	12 (6–20)	0.22	.82
**Word list recall (*n* = 27)**	2 (0–10)	2 (0–9)	0.25	.80
**MRI—Single ROIs (*n* = 31)**				
**Total brain volume [mm^3^]**	1.18 × 10^6^	1.08 × 10^6^	1.81	.070
**White matter**	0.31	0.30	1.90	.058
**Gray matter**	0.34	0.34	0.29	.77
**Temporal left**	4.63 × 10^−2^	4.1 × 10^−2^	**3.57**	**3.59** ×10^−4^**
**Temporal right**	4.74 × 10^−2^	4.84 × 10^−2^	0.11	.91
**Limbic left**	3.02 × 10^−2^	2.80 × 10^−2^	2.67	.0077
**Limbic right**	2.96 × 10^−2^	2.93 × 10^−2^	0.56	.57
**Frontal left**	1.27 × 10^−2^	1.25 × 10^−2^	0.82	.41
**Frontal right**	1.28 × 10^−2^	1.31 × 10^−2^	1.04	.30
**Parietal left**	5.24 × 10^−2^	5.51 × 10^−2^	2.03	.042
**Parietal right**	5.33 × 10^−2^	5.70 × 10^−2^	**3.43**	**5.99** × 10^−4^**
**Hippocampal volume left**	0.21 × 10^−2^	0.21 × 10^−2^	0.29	.77
**Hippocampal volume right**	0.21 × 10^−2^	0.25 × 10^−2^	2.01	.045
**CSF**				
**Amyloid ß1–40 [pg/mL]**	11923	9723	1.31	.19
**Amyloid ß1–42 [pg/mL]**	466.0	920.5	**3.94**	**8.25** × 10^−5^**
**Amyloid‐Ratio**	0.39	0.97	**4.45**	**8.62** × 10^−6^**
**Tau [pg/mL]**	548.0	271.4	**3.62**	**2.90** × 10^−4^**
**Phospho‐tau [pg/mL]**	95.0	47.5	**4.13**	**3.54** × 10^−5^**

### 3.2 Feasibility of the communication assessment

Thirty‐two participants, that is, 96.97% of the sample, completed the *Aachener KOMPASS* questionnaire. One participant, diagnosed with PPA as a result of advanced FTD pathology, was unable to perform the *Aachener KOMPASS* due to impaired verbal communication. In this case, the *Aachener KOMPASS* was performed with a caregiver. There was a consistent agreement between the responses given by the study participants and their caregivers.

On average, it took participants 27 min and 47 s to complete the *Aachener KOMPASS* (range: 15 to 52 min). The FTD group required significantly more time to complete the *Aachener KOMPASS* compared to the AD group (32 min; 25 min; *p* = .039; *zval* = 2.06; *d* = 0.95). The duration for test completion did not correlate with the cognitive state evaluated by the MoCA (*p* = .36 *r*_s  =_  −0.17).

### 3.3 Communication barriers and resources of participants with AD and FTD

**FIGURE 2:**
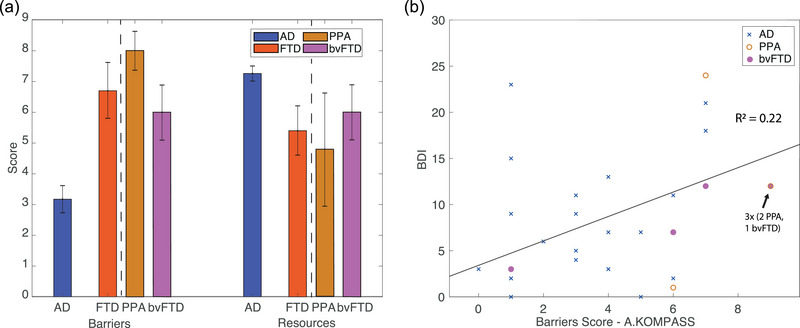
(a) Mean *barriers and resources score* with error bars (representing standard error of the mean) in Alzheimer's disease (AD) and frontotemporal dementia (FTD) (*n* = 10), and subdivision of the FTD group into primary progressive aphasia (PPA) and behavioral variant of FTD (bvFTD). (b) Spearman rank correlation between the *barriers score* and *Beck Depression Inventory* (BDI‐II) score for the AD and FTD groups (*n* = 8/10 with BDI‐II) is shown in different colors; The arrow indicates a data point where three subjects exhibited the same results on both BDI‐II and *barriers score*. Linear regression for the total group is presented (*R* ^2^ = 0.22, *p*‐value = .010).

The communication *barriers score*, as assessed by the *Aachener KOMPASS*, was significantly higher in the FTD group compared to the AD group (*p* = .0015; *zval* = 3.17; *d* = 1.55), indicating a greater prominence of reported communication barriers. In contrast, there was no significant difference in the *resources score* between the two groups (*p* = .14; *zval* = 1.49; *d* = 0.99), as shown in Figure 2a. However, the AD group exhibited higher resources compared to the PPA subgroup within the FTD sample (*p* = .0013; *zval* = 3.22; *d* = 1.19), as illustrated in Figure 2a. A detailed breakdown of the distribution of persons with AD and FTD across the barriers and resources items of the *Aachener KOMPASS* can be found in Table S3. There was a significant association between the *barriers score* and depressive symptoms, as indicated by the BDI‐II score (*p* = .010; *r*_s_ = 0.46), depicted in Figure 2b. Additionally, poorer performance in semantic word fluency was associated with a higher communication *barriers score* (*p* = .025, *r*_s_ = −0.39). However, cognitive status, as measured by the MoCA and the MMSE, did not show a significant correlation with the *barriers score* (MoCA/barriers: *p* = .23 *r*_s_ = −0.22; MMSE/Barriers: *p* = .16, *r*_s_ = −0.25).

For communication resources, no significant correlations were observed with neuropsychological parameters such as the MoCA, MMSE, BDI‐II, and semantic word fluency from the CERAD PLUS test battery.

Regarding the neurodegenerative markers in CSF, a significant correlation was found between total tau levels and the *resources score* (*p* = .022, *r*_s_ = 0.40). An increased *barriers score* was inversely correlated with higher tau and phospho‐tau levels (*p* = .034, *r*_s_ = −0.37; *p* = .036, *r*_s_ = −0.37) and was associated with the amyloid ratio (*p* = .0039, *r*_s_ = 0.49), but not with amyloid β‐1‐42 (*p* = .090; *r*_s_ = 0.30).

The differences in the *barriers score* between the remaining AD patients and FTD group still persisted after excluding the six prodromal AD patients from the AD cohort (*p* = .0049; *zval* = 2.82; *d* = 1.38). This contrasted with the *resources score*, which showed no significant differences (*p* = .16; *zval* = 1.38; *d* = 0.87). This indicates that the differences between the groups were not solely due to an advanced disease state. Furthermore, to rule out that the group differences in communication resources and barriers were driven by the most affected FTD patients, analyses were repeated after excluding the two most affected FTD patients. This resulted in remaining differences in the *barriers score* (*p* = .0091; *zval* = 2.61; *d* = 1.31) while still showing no differences in the *resources score* (*p* = .51; *zval* = 0.65; *d* = 0.45).

### 3.4 Prediction of communication barriers is mainly driven by diagnosis, BDI‐II, and semantic fluency

A multiple regression analysis with LOOCV was consequently performed to predict participants’ communication *barriers score* based on several factors: diagnosis, severity of depressive symptoms measured by the BDI‐II, semantic fluency, relative atrophy, and the global cognitive parameter from the MoCA. The best predictive model included diagnosis, BDI‐II, and semantic fluency as predictors for communication barriers (RMSE = 2.28). In the subsequent ANOVA of this model, diagnosis (FTD or AD), BDI‐II, but not semantic word fluency significantly predicted communication barriers as assessed via the *Aachener KOMPASS* (*t* = 2.20, *p* = .037; *t* = 2.49, *p* = .020; *t* = 0.72, *p* = .48). A detailed overview of this model, represented as “Barriers ∼ Diagnosis + BDI‐II + Semantic fluency,” can be found in Table S4.

### 3.5 Whole brain‐level differences between AD and FTD groups

**FIGURE 3:**
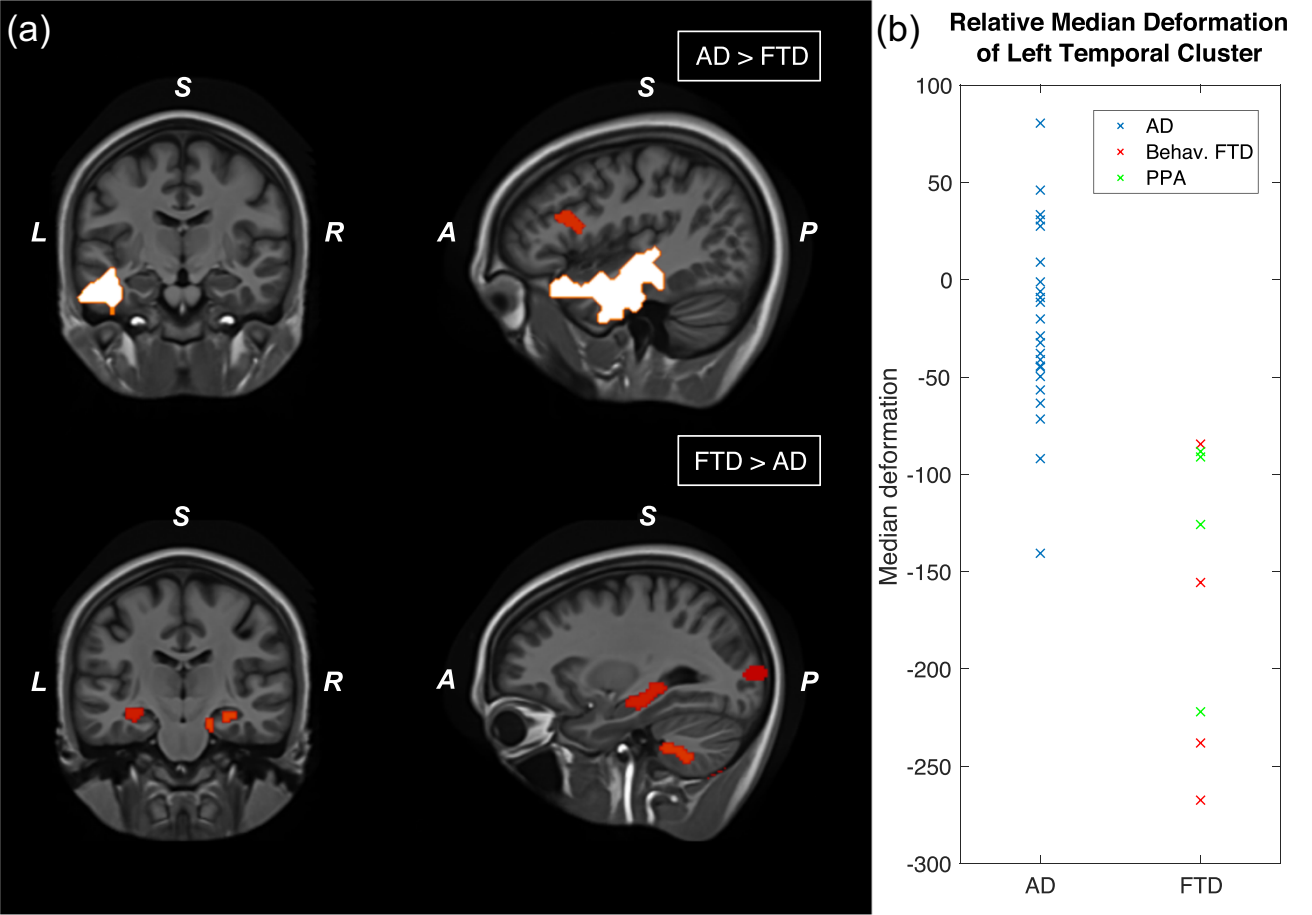
(a) Results of a voxel‐based permutation analysis comparing Alzheimer's disease (AD) and frontotemporal dementia (FTD). In the upper part, the contrast AD > FTD is shown, where clusters surviving correction at the cluster level (*p* < .05) are highlighted in white. In the lower part, the contrast FTD > AD is presented, with uncorrected cluster results in red, which did not survive correction at the cluster level (*p* < .05). Age and sex were included as covariates in the permutation analysis. (b) Illustrates the median deformation values extracted from the left temporal atrophy cluster identified in the AD > FTD contrast, for both AD and FTD subgroups.

To evaluate brain volume differences between the AD and FTD groups, and to understand these differences in relation to communication impairments as assessed by the *Aachener KOMPASS* and other clinical parameters, we conducted a voxel‐wise permutation analysis with the covariates age and sex. The analysis revealed significant differences in the left middle temporal lobe, with the FTD group exhibiting greater volume loss in this region compared to the AD group (corrected at *p*‐value = .05). However, when comparing FTD to AD in the contrast FTD > AD, no volume differences were identified at the corrected *p*‐value threshold of 0.05, but only at an uncorrected level, as shown in Figure 3a. For a comprehensive overview of the contrast clusters converted into MNI (Montreal Neurological Institute) space at the uncorrected level, please refer to Table S2.

### 3.6 Neuronal association of volumetric loss in temporal lobe and communication scoring

**FIGURE 4:**
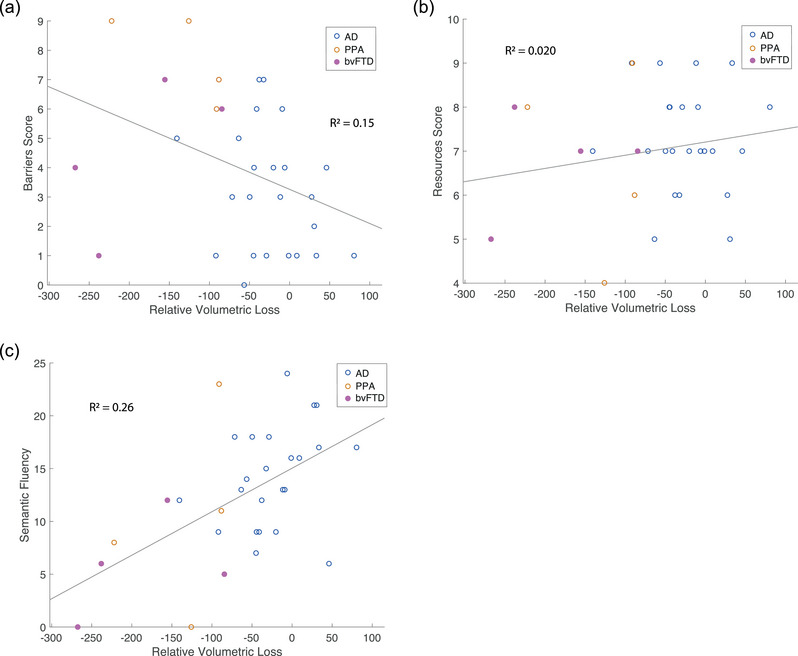
(a) Association between *barriers score* from the *Aachener KOMPASS* and relative volumetric loss (*R* ^2^ = 0.15), that is, relative median deformation and (b) association between resources score and relative volumetric loss (*R* ^2^ = 0.020) and (c) association between semantic fluency and relative volumetric loss (*R* ^2^ = 0.26); All associations were analyzed using Spearman rank correlation.

In a subsequent analysis, we used the temporal cluster identified in the AD > FTD contrast as a regional mask to examine the relationship between brain atrophy and communication scores from the *Aachener KOMPASS* and the semantic fluency in both AD and FTD participants. A voxel‐wise extraction of values of the deformation maps of each individual subject was performed, and the median taken for the estimation of relative volume loss in the referenced cluster. Figure 3b illustrates the distribution of these median deformation values for both AD and FTD participants, further distinguishing between behavioral FTD and PPA patients. Interestingly, we found that regional temporal volume loss was significantly correlated with the communication *barriers score* from the *Aachener KOMPASS* (*p* = .033; *r*_s_ = −0.38) and even more strongly correlated with results from the semantic fluency test of the neuropsychological assessment (*p* = .0038; *r*_s_ = 0.51). However, there was no significant association between the *resources score* and regional temporal volume loss (*p* = .81; *r*_s_ = 0.044), as also shown in Figure 4.

## 4 DISCUSSION

In our study, we explored the communicative impairments of 33 participants with AD and FTD, relating these impairments to brain volumetry from structural high‐resolution MR imaging, neurodegenerative markers from cerebrospinal fluid, and neuropsychological assessment results.

The *Aachener KOMPASS* can be applied as an interview in clinical routine for assessing self‐reported communication barriers and resources in patients with AD and FTD and their caregivers. It can therefore reflect communicative impairment. Due to the possible limited resilience and attention span in these (and other) patients groups, it is essential to use a comprehensible test which can be integrated in clinical exams with a relatively short performance duration which can be integrated in therapeutic sessions (Belleville et al., 2007; Meléndez et al., 2018). Involving caregivers in the assessment process has proven essential, providing additional insights into the extent of communicative impairment and enhancing our understanding of the patients’ social situations and family communication dynamics. Our findings highlighted significant differences in communicative impairment between the AD and FTD groups, as indicated by the *barriers score* in the *Aachener KOMPASS*: Participants with FTD exhibited more communication barriers compared to those with AD. This aligns with our clinical data, where high‐resolution MRI showed greater volume loss in the left temporal regions in patients with FTD. This increased atrophy correlated with higher communication *barriers scores* and decreased semantic word fluency.

Notably, despite these increased barriers, the average completion time of the *Aachener KOMPASS* was 32 min in the FTD sample, demonstrating its feasibility even for patients with more severe communicative impairments. The fact that FTD participants had a longer completion time could be related to their higher communication impairment compared to the AD group.

Previous studies indicate that individuals with FTD, showing increased temporal lobe atrophy compared to those with AD, might experience greater communication barriers in daily life (Hardy et al., 2016; Rousseaux et al., 2010). Interestingly, both AD and FTD patients reported similar levels of communication resources, which may be attributed to a potential reduced awareness of their impairments (Sunderaraman & Cosentino, 2017; Tondelli et al., 2018). Only when compared to the PPA subgroup did the AD group exhibit higher communication resources.

We found no correlation between increased neurodegenerative markers such as total tau and phospho‐tau and the impairment of communication skills. In fact, a higher *barriers score* was associated with lower levels of tau and phospho‐tau. This may reflect the underlying pathology, as FTD patients often have lower tau and phospho‐tau compared to AD patients (Bian et al., 2008; Irwin et al., 2013; Meeter et al., 2017, 2018).

Communication barriers arise from a complex interplay of various deficits, extending beyond language function. These include neuronal circuits in language‐relevant brain regions such as the inferior frontal cortex (e.g., Broca's area) and the superior temporal cortex (e.g., Wernicke's area). Additionally, motor and premotor areas for speech production, the auditory system for language comprehension, and the visual system for reading and interpreting facial expressions during conversation, are crucial. Malfunctions in these areas can lead to increased communication barriers (Friederici & Gierhan, 2013). Specifically, the temporal lobe is involved in speech repetition, complex syntax, language comprehension, semantic processing, and grammar (Brennan et al., 2016; Flinker et al., 2011; Friederici & Gierhan, 2013; Rogalski et al., 2011). Therefore, atrophy in these regions can lead to substantial communication barriers. Several studies tested the specific language skills of persons with dementia, using, for example, the AAT, or tested the communicative skills of persons with aphasia (e.g., via the FCTP). However, the impact of the affected language system on functional communication skills in dementia is less understood. Our findings indicate that communicative impairment, as measured by the *barriers score* from the *Aachener KOMPASS*, correlates with the severity of temporal brain atrophy and is mainly predicted by a model including diagnosis (AD or FTD), BDI‐II, and semantic word fluency. The model is statistically mainly driven by the diagnosis and the BDI‐II as demonstrated in our subsequent ANOVA. This might be due to the predominant role of the factor diagnosis also influencing the factor semantic word fluency, leading to a non‐significant result for the factor semantic fluency in the subsequent ANOVA. One might further argue that the disease state could influence the results on the *barriers score*. However, we found no significant correlations between the *barriers score* and global cognitive scores like the MoCA and MMSE. Similarly, no significant differences in the MoCA and MMSE scores were observed between the FTD and AD groups.

Among the FTD group, five participants were diagnosed with PPA, and five with bvFTD. Among the patients with PPA, three were affected with a semantic variant. In PPA patients, speech production is likely the primary cause of communication barriers (Ash et al., 2019; Mesulam et al., 2014). For the bvFTD and the AD group, there are additional impairments causing communication barriers; bvFTD patients, for instance, often exhibit limited social skills that worsen with progressive brain atrophy, potentially leading to indifferent or withdrawn behavior, low empathy, or even disinhibition in social interactions (Perry et al., 2015). These issues can impair communication and lead to social isolation (Kipps et al., 2009), although bvFTD can also present a language profile similar to svPPA (Hardy et al., 2016). This underscores the importance of distinguishing between dementia forms and understanding the etiologies of communication barriers and their neuropsychiatric consequences.

Our study also demonstrates that communicative impairment, as reflected in the *barriers score*, is associated with the BDI‐II score, indicating depressive symptoms. Participants with more communication barriers exhibited increased depressive symptoms, and the BDI‐II score significantly predicted communication barriers. This finding aligns with existing research linking mental health issues with social isolation (Cacioppo & Hawkley, 2009; Shub et al., 2011) and the impact of social isolation on social and communicative skills (Jootun & McGhee, 2011; Segrin, 1996). It is essential to involve caregivers in the diagnostic process, to capture the mental situation and behavioral disturbances and to consider the social environment of the person with AD or FTD for adapting therapeutic procedures. It highlights the need for interdisciplinary collaboration that address both functional social engagement and communication in dementia and should be considered when interpreting the results of a communication assessment such as via the *Aachener KOMPASS*. Future studies should include further neuropsychiatric assessments beyond the BDI‐II to investigate the interplay between neuropsychiatric symptoms and communication. Additionally, we utilized the global neuropsychological assessment of the CERAD‐Plus, commonly used in clinical settings for diagnosing and monitoring dementia. Including language‐based neuropsychological assessments in future research would be beneficial.

The *Aachener KOMPASS* effectively assesses communication barriers of individuals with AD and FTD, allowing for the identification of their communicative needs. Recognizing these needs and barriers is crucial for setting patient‐oriented therapy goals and enhancing communication resources. However, our study has limitations, including the small sample size of the FTD group, partly due to the rarity of the disease. The fact that both persons with PPA and with bvFTD participated in the study, leads to a certain heterogeneity in this group (but see Heim et al., 2020 vs. Heim et al., 2014 for the role of the left inferior frontal gyrus in both groups). Interestingly, two of our bvFTD patients displayed two of the highest left temporal lobe atrophy clusters in our MR analysis, as did two sv‐PPA patients. The AD group also included prodromal patients who were less clinically affected. Also, the reason for the longer assessment time in the FTD group remains unclear, though it is likely associated with their increased level of communication barriers. Our study did not include a control cohort, focusing instead on applying the *Aachener KOMPASS* in AD and FTD to evaluate communication barriers and resources and their neuronal correlates. Despite varying disease stages, we ruled out the possibility that reduced communication impairment in AD was due to cognitive condition by excluding prodromal AD patients and the most cognitively impaired FTD patients from the analyses. Our findings showed that the differences in the *barriers score* between AD and FTD remained significant even after these exclusions.

The *Aachener KOMPASS* focuses on the characterization of communication barriers and resources, rather than memory impairment, necessitating additional neuropsychological assessment. Some questions in the *Aachener KOMPASS* indirectly relate to cognitive impairment, such as the ability to follow a conversation, though.

A major strength of our study is demonstrating the link between communication barriers in AD and FTD as assessed by the *Aachener KOMPASS* and their clinical correlations. This offers potential for developing therapeutic strategies based on the reported communicative impairment and resources. Exploring associations with other communication and aphasia assessments could provide further insights. Further studies on the functional communication skills of persons with different dementia forms and larger, preferably multicentric, samples, especially with different forms of FTD, are needed to further differentiate between pathologies. This approach will support clinicians in offering individualized and tailored therapy strategies.

## 5 CONCLUSIONS

In the present study, we assessed communication barriers and resources in AD and FTD via the *Aachener KOMPASS*, a questionnaire on daily functional communication in form of an interview for patients and their relatives. We found that communication barriers are more prominent in FTD and are clinically associated with depressive symptoms, left temporal lobe atrophy, and reduced semantic fluency. Unlike communication barriers, reported communication resources did not differ between the AD and FTD groups and were not reflected at the neuronal level assessed via MRI or neuropsychological assessments. This highlights the specificity of communication barriers for distinct brain pathologies. Communicative resources can still be relevant for setting therapeutic goals, though, and when regarding subgroups, as shown in our sample. Furthermore, it seems to be especially important to consider neuropsychiatric symptoms such as depression, as indicated by the BDI‐II score, which strongly influences communication barriers and is therefore highly relevant for the development of therapeutic strategies. The *Aachener KOMPASS* serves in this context as a clinically applicable tool for speech language therapists, clinicians, and neuropsychologists to assess the severity of communication barriers in the lives of individuals with AD and FTD, aiding in understanding the neurobiology of impaired communication. The assessment of communication barriers and resources—also via other conceptualized questionnaires or interviews—is therefore clinically important, also for an optimization of speech therapy adapted to the clinical status and the communicative needs for improving the quality of life of the affected persons. Our findings contribute to a better understanding of the neuroanatomical and neurofunctional characteristics of impaired communication in AD and FTD.

## AUTHOR CONTRIBUTIONS

KR, SH, JBS, BF, AH contributed to the study design and conzeptualization. JM performed the Aachener KOMPASS. AH and SR performed the MR processing. AH and JM performed the statistical analyses and wrote the first draft of the manuscript. All authors advised, edited and reviewed the manuscript.

## CONFLICT OF INTEREST STATEMENT

The authors declare no conflicts of interest.

### PEER REVIEW

The peer review history for this article is available at https://publons.com/publon/10.1002/brb3.3420.

## Supporting information

Supplementary Table S1: Overview of the FTD sample: Note that for subject FTD_3, the BDI‐II (Beck Depression Inventory‐II) data are not available because the subject did not complete the entire neuropsychological assessment, due to difficulties in understanding the test instructions. Subject FTD_9 was too impaired to understand the test instructions and was consequently scored with 0 points for both the MoCA (Montreal Cognitive Assessment) and the MMSE (Mini‐Mental State Examination).
Supplementary Table S2: Peak coordinates in MNI space of VBM clusters of contrasts AD > FTD and FTD > AD from a permutation analysis with age and sex as covariates. n = 2000 permutation at p < 0.05. uncorrected at cluster level. Significant cluster corrected at p < 0.05 at cluster level is presented in bold print.
Supplementary Table S3: This table lists the items of the Aachener KOMPASS along with the number of participants (n) with Alzheimer's disease (AD) and frontotemporal dementia (FTD), including subcategories of primary progressive aphasia (PPA) and behavioral variant FTD (bvFTD), who identified each item as either a barrier or a resource. It details the barriers score and resources score, with nine categories for each. Open‐ended questions in the questionnaire are marked with an asterisk (*).
Supplementary Table S4: This table presents the results of the Leave‐One‐Out Cross‐Validation (LOOCV) multiple regression analysis. The model, represented as ‘Barriers ∼ Diagnosis + BDI‐II + Semantic Fluency’, was identified as the best fitting model with a Root Mean Square Error (RMSE) of 2.28.

## Data Availability

Data may be made available on reasonable request to the corresponding author.
