# Supplementary material for: Communicative impairment and its neural correlates in Alzheimer's disease and frontotemporal dementia
Source: Brain Behav. 2024 Mar 17;14(3):e3420. doi: 10.1002/brb3.3420 (PMC10945087; doi:10.1002/brb3.3420)
Supplement: Supplementary file 1 — Supplementary Table S1: Overview of the FTD sample: Note that for subject FTD_3, the BDI‐II (Beck Depression Inventory‐II) data are not available because the subject did not complete the entire neuropsychological assessment, due to difficulties in understanding the test instructions. Subject FTD_9 was too impaired to understand the test instructions and was consequently scored with 0 points for both the MoCA (Montreal Cognitive Assessment) and the MMSE (Mini‐Mental State Examination). Supplementary Table S2: Peak coordinates in MNI space of VBM clusters of contrasts AD > FTD and FTD > AD from a permutation analysis with age and sex as covariates. n = 2000 permutation at p < 0.05. uncorrected at cluster level. Significant cluster corrected at p < 0.05 at cluster level is presented in bold print. Supplementary Table S3: This table lists the items of the Aachener KOMPASS along with the number of participants (n) with Alzheimer's disease (AD) and frontotemporal dementia (FTD), including subcategories of primary progressive aphasia (PPA) and behavioral variant FTD (bvFTD), who identified each item as either a barrier or a resource. It details the barriers score and resources score, with nine categories for each. Open‐ended questions in the questionnaire are marked with an asterisk (*). Supplementary Table S4: This table presents the results of the Leave‐One‐Out Cross‐Validation (LOOCV) multiple regression analysis. The model, represented as ‘Barriers ∼ Diagnosis + BDI‐II + Semantic Fluency’, was identified as the best fitting model with a Root Mean Square Error (RMSE) of 2.28. [file BRB3-14-e3420-s001.docx]

**Supplementary**

| Subject ID |  | **Gender** | **Barriers score** | **Resources score** | **BDI-II** | **MOCA** | **MMSE** |
| --- | --- | --- | --- | --- | --- | --- | --- |
| FTD_1 | Behav FTD | M | 6 | 7 | 7 | 19 | 28 |
| FTD_2 | nf-avPPA | F | 7 | 6 | 24 | 21 | 23 |
| FTD_3 | Behav FTD | F | 4 | 5 |  | 11 | 19 |
| FTD_4 | Behav FTD | M | 7 | 7 | 12 | 18 | 28 |
| FTD_5 | svPPA | M | 9 | 1 | 12 | 2 | 4 |
| FTD_6 | Behav FTD | M | 9 | 3 | 12 | 21 | 27 |
| FTD_7 | svPPA | F | 9 | 8 | 29 | 17 | 23 |
| FTD_8 | Behav FTD | F | 1 | 8 | 3 | 12 | 21 |
| FTD_9 | svPPA | F | 9 | 0 |  | 0 | 0 |
| FTD_10 | nf-avPPA | M | 6 | 9 | 1 | 28 | 30 |

*Supplementary Table S1: Overview of the FTD sample: Note that for subject FTD_3, the BDI-II (Beck Depression Inventory-II) data are not available because the subject did not complete the entire neuropsychological assessment, due to difficulties in understanding the test instructions. Subject FTD_9 was too impaired to understand the test instructions and was consequently scored with 0 points for both the MoCA (Montreal Cognitive Assessment) and the MMSE (Mini-Mental State Examination).*

| Contrast | MNI-coordinates x y z | t-value |  |
| --- | --- | --- | --- |
| AD > FTD | **-45 1 -25** | **4.43** |  |
|  | -43 23 17 | 4.05 |  |
|  | -16 -50 31 | 4.80 |  |
| FTD > AD | 34 -17 -20 | 4.15 |  |
|  | -31 -23 -15 | 4.25 |  |
|  | -15 -56 15 | 3.85 |  |
|  | -31 -49 -48 | 3.68 |  |
|  | -1 -66 -40 | 2.34 |  |
|  | 20 -64 47 | 3.02 |  |

*Supplementary Table S2: Peak coordinates in MNI space of VBM clusters of contrasts AD>FTD and FTD>AD from a permutation analysis with age and sex as covariates. n = 2000 permutation at p < 0.05. uncorrected at cluster level. Significant cluster corrected at p < 0.05 at cluster level is presented in bold print.*

**Supplementary information on the Aachener KOMPASS**

For the Aachener KOMPASS, normative data are available from a sample of *n* = 95 healthy participants. These data include item ratings for 'personal relevance' and 'frequency of occurrence in daily life' for each item. The sample is divided into two age groups: 'aging persons' (60-75 years; *n* = 61) and 'older' persons (> 75 years; *n* = 34). Since the items of the Aachener KOMPASS can be used individually, the item set may be tailored for clinical purposes, allowing for adaptation to different neurological cohorts. Accordingly, the KOMPASS was revised in 2017 to create a short version suitable for individuals with cognitive impairments (Heim, 2020; Rembeck, 2017). This shorter version comprises 17 multiple-choice questions and three open-ended questions.

The Aachener KOMPASS assessment begins with the examiner collecting general demographic information about the participants, such as age, education, gender, employment, and family situation. This is followed by questions concerning their use of media devices, types of conversation partners, communicative behavior, and listening, speaking, reading, and writing skills. 'Barriers' in the context of the KOMPASS refer to obstacles in communication, such as difficulties in phone conversations or challenges in following unfamiliar topics during discussions. In contrast, 'resources' pertain to positive or reinforcing aspects of communication, like the use of media devices, or engaging in writing or reading during leisure time.

| Items: Barriers Score | | AD  (n=23) | FTD (n=10) | PPA (n=5) | bvFTD (n=5) |
| --- | --- | --- | --- | --- | --- |
| 1  2  3  4  5  6  7  8  9* | Difficulties on the phone  Conversations in groups  Conversations with strangers  Solving misunderstandings  Changing topic in a conversation  Getting involved in discussions  Following a conversation  Following unfamiliar topics  Changes in communicative behavior | 12  6  6  7  7  10  11  11  8 | 8  7  8  6  9  8  8  6  7 | 4  4  5  5  5  5  4  3  5 | 4  3  3  1  4  3  4  3  2 |
| Items: Resources Score | |  |  |  |  |
| 1  2  3  4  5  6  7  8  9* | Number of conversation partners  Writing during leisure time  Writing in daily situations  Reading during leisure time  Reading in daily situations  Usage of media devices  Hobbies  Situations with conversations  Changes in last 5 years  (yes = barrier; no=resource) | 23  20  21  22  21  20  9  18  13 | 9  6  7  7  7  8  1  5  4 | 4  3  3  3  3  3  1  2  2 | 5  3  4  4  4  5  0  3  2 |

*Supplementary Table S3: This table lists the items of the Aachener KOMPASS along with the number of participants (n) with Alzheimer's disease (AD) and frontotemporal dementia (FTD), including subcategories of primary progressive aphasia (PPA) and behavioral variant FTD (bvFTD), who identified each item as either a barrier or a resource. It details the barriers score and resources score, with nine categories for each. Open-ended questions in the questionnaire are marked with an asterisk (*)*

**Barriers~ Diagnosis + BDI-II + Semantic fluency**

**RMSE 2.284737**

| *Coefficients:* | |  |  |  |
| --- | --- | --- | --- | --- |
|  | **Estimate** | **Std Error** | **t-value** | **p-value** |
| (Intercept) | 5.28 | 1.25 | 4.21 | 0.000285*** |
| Diagnosis | -2.21 | 1 | -2.2 | 0.0370* |
| BDI-II | 0.13 | 0.05 | 2.49 | 0.0196* |
| Semantic | -0.053 | 0.073 | -0.72 | 0.477 |

*Supplementary Table S4: This table presents the results of the Leave-One-Out Cross-Validation (LOOCV) multiple regression analysis. The model, represented as 'Barriers ~ Diagnosis + BDI-II + Semantic Fluency', was identified as the best fitting model with a Root Mean Square Error (RMSE) of 2.28.*
